# Developing a Risk Governance Framework on Radiological Emergency, Preparedness, and Response for Emergency Responders: Protocol for a Mixed Methods Study

**DOI:** 10.2196/25877

**Published:** 2021-08-13

**Authors:** Anita Abd Rahman, Rosliza Abdul Manaf, Poh Ying Lim, Subapriya Suppiah, Muhammad Hanafiah Juni

**Affiliations:** 1 Department of Community Health Faculty of Medicine and Health Sciences Universiti Putra Malaysia Seri Kembangan Malaysia; 2 Department of Radiology, Faculty of Medicine and Health Sciences Universiti Putra Malaysia Seri Kembangan Malaysia

**Keywords:** emergency, preparedness, radiological, risk governance, risk practices

## Abstract

**Background:**

Risk governance involves processes and mechanisms to understand how risk decisions are taken and executed. This concept has gained a reputation over time as being essential for emerging comprehensive management that defines the success of an organization. While guiding documents that explain the use of risk management related to nuclear safety and security are available worldwide, few locally conducted studies have explained risk governance practices in areas where hazard usage is known, such as in radiological emergencies.

**Objective:**

This paper describes a protocol that was used to determine several factors that influence emergency responders’ perceptions toward radiological risk practices and visualize the risk radiological framework for emergency preparedness and response.

**Methods:**

A mixed methods study with a convergent design was performed. A qualitative analysis was performed using a case study approach where 6 key informants were purposely sampled for in-depth interview, and a cross-sectional study involving a self-administered questionnaire was conducted among approximately 260 emergency respondents from national regulatory, research, and services organizations. NVivo (version 12, QSR International) was used to analyze the interview transcripts and emerging themes were identified through abductive coding. Simultaneously, multiple logistic regression analysis was used to determine significant predictors that form the equation model.

**Results:**

The study is still underway. Qualitative findings were based on transcript-coding that informed the relevant thematic analysis, while statistical analyses including multiple logistic regression analysis measured the adjusted odds ratio of significant variables for the equation model. The study is expected to conclude in late 2021.

**Conclusions:**

Important emerging themes and significant factors that are related to the emergency responders’ perceptions regarding radiological governance practices were determined through the convergent design. This potentially facilitated the development of a plausible radiological risk governance framework. Furthermore, our results will provide key insights that can be used in future studies.

**International Registered Report Identifier (IRRID):**

DERR1-10.2196/25877

## Introduction

### Background

The philosophy of governance, existing since medieval times, has come a long way. Far from being perfect, practices have evolved from a simplistic stewardship theoretical approach to a more complex dynamic model and may continue to develop in parallel with globalization [1]. Despite its popularity in the corporate, business, and economic sectors in certain fields, the application of governance may not be well-known or is unrecognized. However, previous studies have shown that governance plays an integral part in numerous organizational managements and is considered the main foundation for organizational sustainability.

One particular area where governance is gaining popularity is within working sectors that use certain materials that are known to be hazardous to health and have the potential to cause disasters if not managed properly. For example, in the medical fraternity, the concept of governance has led to the development of a documented manual by the World Health Organization, entitled “Rapid Risk Assessment of Acute Public Health Events,” which serves as a guide for a systematic process of rapid and defensible decision-making to deal with hazardous events of a biological or chemical nature and re-emerging diseases [2]. This manual also addresses multidisciplinary players and stakeholders in prevention and control, including effective communication to improve national preparedness. Similarly, this concept was adopted by Schmidt et al [3] for better and more effective management to combat challenges in vector-borne diseases. It has been observed that when governments or organizations develop new services in combatting disasters, other uncertainties such as financial risks, time risks, or psychological risks may arise and should be considered. Conscious management of the transparent process can promote a more successful service-related outcome [4].

In the context of health and safety, the concept of risk management involves valued judgments that reflect the probability and consequences of the occurrence of an event [5], which is a common misnomer to risk governance. Under these circumstances, risk management does not equate to risk governance as it may have relatively minimal focus on other areas such as financial and legal sectors and interaction of the Internet of Things, which rely on a clear and robust code of practice for the entire management [6]. Therefore, the term “risk governance” has been explicitly described by the International Risk Governance Council as a nonprofit organization that facilitates a better understanding of risks and their scientific, political, social, and economic contexts and translates the core principles of governance to the context of risk and risk-related decision-making of an organization [7,8].

To establish a system in radiological emergency preparedness and response (EPR), the International Atomic Energy Agency (IAEA) has developed a few documents that recommend what forms the basis of and the requirements for an adequate level of preparedness and response for a nuclear or radiological emergency. In addition, these documents have also described the necessary implementation of specific safety requirements; for example, guidelines on a coordinating mechanism and communicating with the public in emergency preparedness and response considering certain circumstances. All these can be seen as the gold-standard guide for any of the IAEA member states to develop its own radiological governances that encompass all the requirements. However, local studies have mainly focused on the characteristics of EPR itself from an operational perspective, but few studies have implemented a governance perspective.

It is currently speculated that the available local radiological framework focuses on the legislative and organizational components with minimum information on risk practices and community involvement. It was also revealed that under the Radiological Emergency Preparedness and Response Training and Capability Development in Southeast Asia, certain countries still had issues related to radiological EPR, where recommendations were made to improve the integration of radiological responses into an all-hazards approach and related interagency interoperability [3].

Thus, having a proper framework encompassing relevant factors, areas, and people is key to success especially in radiological EPR, and it is speculated that such studies have been deemed necessary to evaluate local governance practices that are in place for radiological EPR management. Here we describe a protocol used to determine relations among sociodemographic, occupational, cultural, social, ethical values, decision-making, and trust factors that influence emergency responders’ perceptions toward radiological risk practices. Additionally, this protocol would help researchers develop a more customized radiological risk governance framework.

### Underpinning Theory

Two major components that constitute governance are system and people; accordingly, this study adopted 2 types of theoretical models. The first model is the Social Action Theory mooted by one of the pioneer sociologists Max Weber in the early 1900s, which examines the actions of people in the context of meanings assigned to them and their relationship with the actions of others. This is important in determining one’s perception of risk as it is based on subjective assessment of an individual’s frame of reference developed over time, with respect to risk management. This influences the evaluation of the probability of a specified type of accident occurring and how concerned a person is with the consequences.

The second theory is based on the risk governance framework developed by the International Risk Governance Council—a Switzerland-based private, independent, nonprofit foundation established in 2003—and represents a system that uses the following 5 elements [9]:

1. Risk preassessment: early warning and “framing” of risk to provide a structured definition of the problem to describe how it is framed by various stakeholders and how it can be managed optimally.
2. Risk appraisal: combining a scientific risk assessment (of the hazard and its likelihood) with a systematic concern assessment (of public concerns and perceptions) to provide a knowledge base for subsequent decisions.
3. Characterization and evaluation: scientific data and a detailed understanding of risk-affected societal values are used to evaluate the risk as acceptable, tolerable (requiring mitigation), or intolerable (unacceptable).
4. Risk management: actions and remedies required to avoid, reduce, transfer, or retain the risk.
5. Risk communication: how stakeholders and civil society understand the risk and participate in the process of risk governance.

The use of these 2 theories provided insight into the research conceptual framework.

## Methods

**Figure 1:**
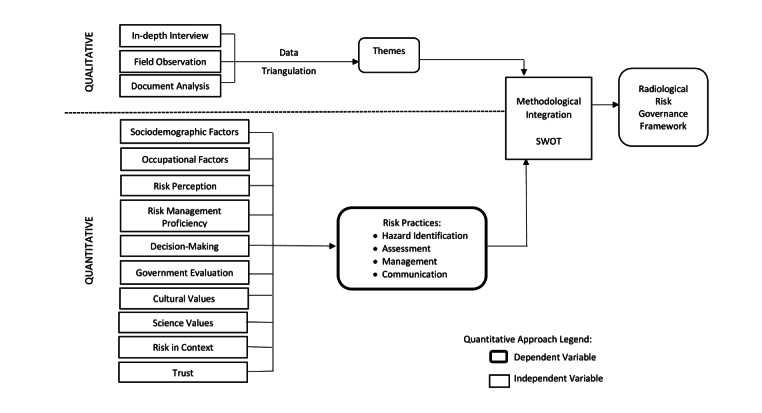
Conceptual framework of the study. SWOT: strengths, weaknesses, opportunities, and threats.

This was one of the earlier proposed local studies that is focused on radiological risk governance practices, and the application of both quantitative and qualitative assessments is important to further support the evaluation of risk governance that is in place for the management of radiological technology. Furthermore, the philosophical assumption of mixed methods studies is often referred to as the third methodological approach that has attracted both academics and researchers who were primarily either positivists or interpretivists [10]. Based on the theories that were considered, the conceptual framework of our study is shown in Figure 1.

Klang Valley was selected as the study location as it is the prime area where most radiological applications and activities are concentrated and where radiological EPR will be activated (if it occurs). Table 1 illustrates the mixed methods approach in terms of its aim, design, extension, sample size, instrument to be used, analysis, and interpretation.

The selection of respondents/informants was based on the following criteria where those aged ≥18 years old, those working as emergency responders at an organization, those involved in radiological governance policy-/decision-making, or those having experience related to radiological risk governance were eligible to participate in the study. In contrast, those who were absent during study data collection (eg, international travel/training) and those who refused to participate in the study were excluded.

**Table 1:** Characteristics of the mixed methods approach.

Characteristics	Quantitative research	Qualitative research
Aim	Provide an understanding of the research questions	Proves research hypothesis
Design	Cross-sectional study	Case study approach
Extension	Breath view	In-depth view
Sample size	Sample size is calculated using a sample size formula for a number to estimate prevalence on the basis of proportion [11] and with a known (finite) population of 500 emergency responders within the study area [12]. Total=260 respondents.	Generally smaller; until achieving a saturation point
Sample selection	Simple random sampling	Purposely involving the 7 governmental agencies
Instrumentation	Standard questionnaire	In-depth interview
Analysis and interpretation	Through statistical analysis including bivariate analysis as well as correlation and prediction using multiple logistic regression analysis	Identify research themes
Reporting guidelines or protocol	Strengthening the Reporting of Observational Studies in Epidemiology checklist	Consolidated criteria for reporting qualitative research checklist

The qualitative approach used an in-depth interview technique through a semistructured interview protocol that included the following core questions:

1. What is the general governance’s framework in radiological EPR?
2. How does the emergency responder perceive the use of current governance’s framework in radiological EPR?
3. How to improve the current governance’s framework in radiological EPR?

A total of 6 key informants were purposely chosen as they represent each responsible organization that fit with the aforementioned selection criteria. The entire interview was audiotaped, and transcripts were analyzed using NVivo (version 12, QSR International) which provided the basis for thematic analysis.

The quantitative method utilized a standard questionnaire adopted from previous risk governance studies on climate change, radiation emitted from mobile phones, and radioactive waste [13,14]. This questionnaire has been validated among 1547 respondents through face-to-face interviews and was widely accepted as a reliable method (Cronbach α on reliability analysis ranging .58-.89). The 5-point Likert Scale questionnaire aimed to provide hypothetical reasoning in the field of risk management, which encourages theoretical understanding. A precalculated sample of 260 respondents were administered a self-administered questionnaire. Independent variables comprising both continuous and categorical data were input in the statistical analysis using IBM SPSS software version 25. Logistic regression analysis was used to exhibit the association between the independent variables and radiological risk practices as the dependent variable. Based on simple logistic regression analysis, variables with significant *P* values of <.25 were selected for subsequent multiple logistic regression analysis to determine predictors with significant *P* values of <.05 regarding radiological risk practices.

Finally, research ethic approvals were gained from 2 organizations, namely the Medical Research Ethics Committee at Universiti Putra Malaysia (UPM/TNCPI/RMC/ JKEUPM-2018-014) and the Medical Research Ethics Committee of the Ministry of Health, Malaysia (NMRR-18-1922-40686). Informed formal consent was also obtained from each respective organization where the respondents were sampled from.

## Results

The qualitative result was based on interviews from 6 key informants describing the relevant thematic analysis, while quantitative data were presented as descriptive statistics and analyzed using multiple logistic regression analysis, which yielded adjusted odds ratios for significant variables for the equation model. The hypothesized relationship was depicted in a multiple regression equation as follows:

Odds (radiological risk practices) = b_1_x_1_ + b_2_x_2_ + … + b_n_x_n_ + c

**Figure 2:**
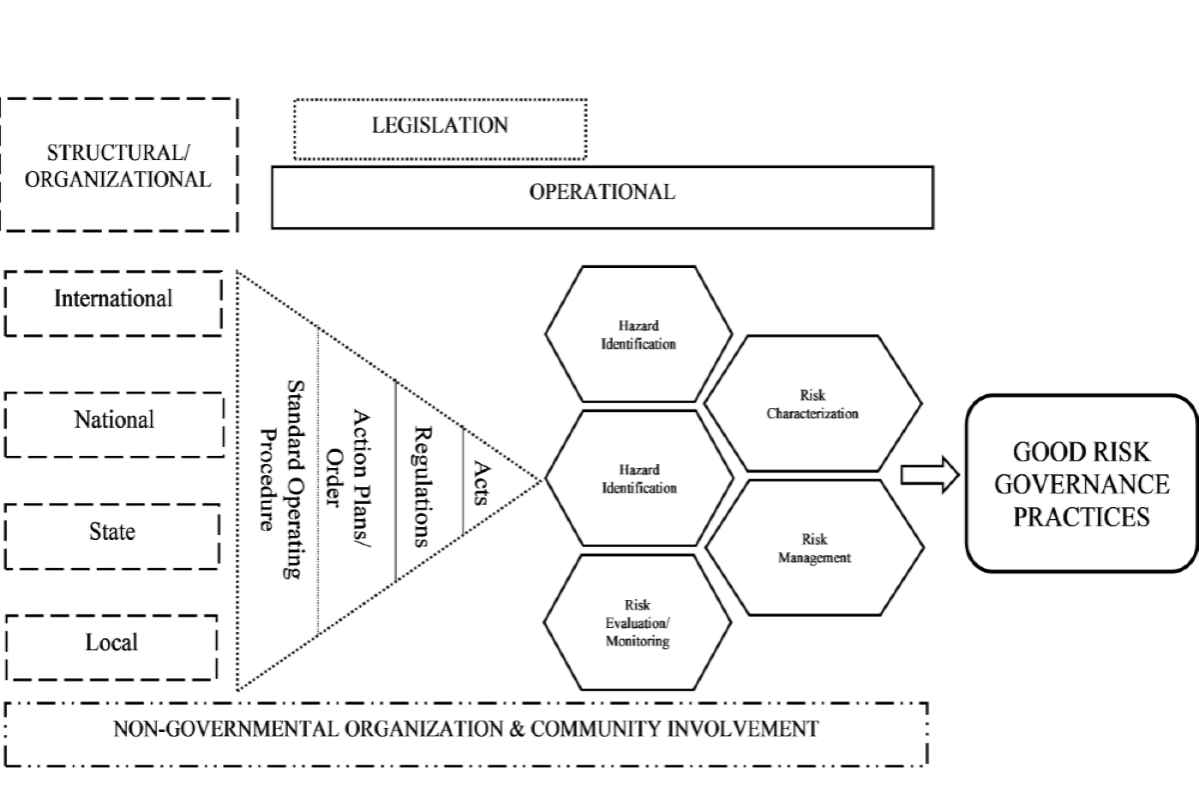
Proposed risk governance framework on radiological emergency, preparedness, and response for emergency responders. SWOT: strengths, weaknesses, opportunities, and threats.

Converging the 2 findings in the form of a joint display table facilitated further interpretation among various factors and addressed all research objectives as data integration is a key element for mixed methods* *analysis. Based on all findings, a proposed radiological risk governance framework was tabled out with a preliminary version. Furthermore, the framework was also aligned with the national sustainable development goals to be cohesive, transparent, accountable, and relevant with time [15]. Figure 2 shows the preliminary framework. The study is still underway and is expected to conclude in late 2021.

## Discussion

### Principal Findings

This paper describes a protocol that was used to address governance concepts and practices, particularly in the field of radiological EPR. Through this convergent research design, this study aimed to understand and evaluate the current governance, with quantitative methods, using statistical analysis that includes relationship testing. The hypothesized significant relationship between the studied factors and emergency responder perception on radiological governance potentially revealed significant factors such as risk perception, risk management proficiency, organization, and government involvement, and analysis was depicted in a model that represented the hypothesized causal/predictive relations. Through in-depth interviews, the qualitative approach possibly reveals explanatory and textual emerging themes that may not have been discovered before, and this can be viewed as a part of an ongoing process that helps improve performances for current and future management to achieve desired outcomes.

It is known that risk governance plays a pertinent role in the technological use of radioactive material in various fields because of its potential for global impact. The Atomic Energy Licensing Act was passed in 1984 [16] owing to the rapid development of the applications of radioactive material and activities in Malaysia, which require effective control, enforcement, and ensuring of safe and peaceful use. Furthermore, National Security Council directive 20 emphasizes the policy and mechanism of an integrated management system for disaster and relief management on land, which includes radiological emergencies before, during, and after disaster stages as well as determining roles and responsibilities of various agencies involved in disaster management [17]. Similarly, several international documents from the IAEA have explained the safety standards in terms of fundamentals and requirements that are necessary for preparedness and response for a nuclear or radiological emergency [18,19] right until the termination of the emergency response [20]. Simultaneously, a reference manual on the generic procedures for the initial response toward a radiological accident by each organization and different phase responses is also available from among the IAEA technical documents [21]. Regarding communication, this component should also concur with international recommendations for a transparent and accurate provision of official information as well as having a practicable coordinated response [22].

### Limitations

The involvement of multiple stakeholders from several organizations that are currently involved in radiological EPR, such as enforcement agencies, the police, armed forces, firefighters, medical teams, and university and research centers, while potentially adding more data value, took a long time and required plenty of resources and support. Another challenge was related to data integration and the finalization of interpretive findings as there are still limited resources that can support an overall comprehensive governance framework.

### Conclusions

Important emerging themes and significant factors related to emergency responders’ perceptions on radiological governance practices were determined through the convergent design. This potentially facilitated the development of a plausible radiological risk governance framework to strengthen the existing process as this is in tandem with good governance practice that promotes continuous improvement for prevention and control in radiological emergency, preparedness, and response.

## Abbreviations

EPR: Emergency Preparedness and Response
IAEA: International Atomic Energy Agency
